# Extract of *Plantago asiatica* L. Seeds Ameliorates Hypertension in Spontaneously Hypertensive Rats by Inhibition of Angiotensin Converting Enzyme

**DOI:** 10.3389/fphar.2019.00403

**Published:** 2019-04-30

**Authors:** Ren-Chao Tong, Meng Qi, Qi-Ming Yang, Peng-Fei Li, Dan-Dan Wang, Ji-Ping Lan, Zheng-Tao Wang, Li Yang

**Affiliations:** ^1^The Ministry of Education Key Laboratory for Standardization of Chinese Medicines and the State Administration of Traditional Chinese Medicine Key Laboratory for New Resources and Quality Evaluation of Chinese Medicines, Institute of Chinese Materia Medica, Shanghai University of Traditional Chinese Medicine, Shanghai, China; ^2^Institute of Interdisciplinary Integrative Medicine Research, Shanghai University of Traditional Chinese Medicine, Shanghai, China

**Keywords:** antihypertenisve, *Plantago asiatica* L. seeds, angiotensin I-converting enzyme, spontaneously hypertensive rat, organ damage, phenylethanoid glycosides

## Abstract

*Plantago asiatica* L. seeds is a common folk medicine with a long history of medical use in China because of its antipyretic, diuretic, and expectorant properties. It has been applied to treat hypertension clinically due to its diuresis, however, its efficacy and mechanisms on anti-hypertension has not been reported yet to our knowledge. In this study, we investigated the antihypertensive effect and underlying mechanisms of *P. asiatica* L. seeds extract (PASE) in spontaneously hypertensive rat (SHR). Male SHRs were treated with 2.5 mg/kg of fosinopril (FOS) and 400 mg/kg of PASE orally per day for once or 12 weeks. SHR or Wistar-Kyoto rats (WKY) receiving vehicle (distilled water) was used as control. The results demonstrated systolic, diastolic, and mean blood pressures (SBP, DBP, and MBP) were significantly lowered after single and long-term intragastric administration of PASE. The cardiac and aortic index and collagen accumulation were improved in the PASE group compared with the SHRs group. Meanwhile, PASE treatment remarkably reduced urine total protein, the ratio of serum urea nitrogen to serum creatinine, and increased serum potassium. The levels of serum angiotensin I (Ang I), angiotensin II (Ang II), the ratio of Ang II to Ang I, and aldosterone (ALD) were lowered after treatment of PASE. Besides, PASE and its major active constituents of phenylethanoid glycosides, including isoacteoside, plantamajoside and acteoside, were found to effectively inhibit angiotensin-converting enzyme (ACE) activation *in vitro*. These findings suggest that PASE has the antihypertensive effect that may involve a mechanism of ACE inhibition and simultaneously protect organ damage against hypertension.

## Introduction

Hypertension is one of the major threats for global human health, which could induce a series of damages to brain vessel, heart, and kidney (WHO, 2013). Genetic inheritance, aging, bad lifestyle, long-term mental stress, and other diseases such as obesity and diabetes could be the possible causes of hypertension. Hypertension is closely related to the function of renin–angiotensin–aldosterone system (RAAS), a hormone system that plays an essential role in the regulation of the cardiovascular development, electrolyte balance, and blood pressure (Te et al., 2015).

Nowadays, many chemical drugs are used in blood pressure control with outstanding pharmacological effects, however, they have non-negligible side effects such as headache, asthma, and loss of plasm potassium, etc. In recent years, traditional herbal medicines have attracted special attention on hypertension treatment and new drug development since they contain various natural products with reported antihypertensive activities, such as flavonoids, terpenes, alkaloids, and phenolic compounds (Maione et al., 2013; Bai et al., 2015). However, it is a persistent challenge to develop a traditional Chinese medicine because of its complex composition and undefined mechanism. *Plantago asiatica* L. seed, also known as Plantaginis Semen, has been used as medicine and a food plant with a long history in China for antipyretic, diuretic, and expectorant purposes. Previous studies have shown that *P. asiatica* L. seed contains polysaccharides, phenylethanoid glycosides, iridoids, flavonoids, triterpenes, et al. (Huang et al., 2014; Qi et al., 2015; Wang et al., 2016), which account for a variety of properties such as immunomodulatory, anti-oxidation, anti-inflammation, liver protection, facilitating defecation, improving lipids/glucoside metabolism, and so on (Xu et al., 2004; Hannan et al., 2006; Huang et al., 2009; Geng et al., 2010; Yin et al., 2010; Lim et al., 2013; Yang et al., 2017). Nevertheless, the effective components and mechanism of *P. asiatica* L. seed on modern hypertension treatment are still unclear. In the study presented here, we experimentally confirmed that the extract of *P. asiatica* L. seeds (PASE) had significant inhibitory activity on ACE *in vitro with the* major active constituents of phenylethanoid glycosides including isoacteoside, plantamajoside and acteoside. We also demonstrated that *P. asiatica* L. seeds can reduce blood pressure and protect heart, aorta, and kidney in rat models, indicating the potential use of *P. asiatica* L. seed in hypertension treatment.

## Materials and Methods

### Plant Material and Extraction

Dried seeds of *P. asiatica* L. were purchased from Kangqiao Pharmaceutical Co., Ltd. (Shanghai, China). The identification was confirmed by Lihong Wu, Institute of Chinese Materia Medica, Shanghai University of Traditional Chinese Medicine, China. The seeds were powdered before being immerged into 10 times its volume of ethanol-water (60:40) solution overnight and then reflux extracted for 3 times, each time for 2 h. Filtrates were combined, concentrated under reduced pressure and freeze-dried to provide extract of *P. asiatica* L. seed (PASE). The extraction yield was about 16% (g/g). The extract was stored at −20°C and dissolved with distilled water before being administrated to the rats.

### Ultra-Performance Liquid Chromatography-Mass Spectrometry (UPLC-MS) Analysis

The analysis of PASE was performed on an Acquity UPLC system (Waters, United States) combined with an Acquity Synapt G2 QTOF tandem mass spectrometer (Waters, United Kingdom). An Acquity UPLC BEH C18 RP column (1.7 μm, 100 mm × 2.1 mm i.d.; Waters, United States) was employed for the chromatographic separation with the column temperature at 45°C. The mobile phase consisted of 0.1% formic acid in deionized water (mobile phase A) and acetonitrile (mobile phase B) at a flow rate of 0.3 ml/min with the following gradient: 0–4 min, 15% B; 4–7 min, 15–17% B; 7–9 min, 17–23% B; 9–14 min, 23–50% B; 14–28 min, 50–95% B, and 28–30 min, 5% B for equilibration of the column. In the MS analysis, the ESI source was operated in both positive (ESI+) and negative (ESI-) ionization mode with the following parameters: capillary voltage, −2.5 kV (ESI−) or +3 kV (ESI+); sample cone, 30 V; extraction cone, 4.0 V; source temperature, 120°C; desolvation temperature, 350°C; cone gas (nitrogen) flow, 50 L/h; and desolvation gas (nitrogen) flow, 600 L/h. Instrumental control and data collection and processing were conducted by MassLynx V4.1 software (Waters Corp., Milford, MA, United States).

### Experimental Animals

Male spontaneously hypertensive rats (SHR, 8–10 weeks old, SPF) and Wistar -Kyoto rats (WKY, 8–10 weeks old, SPF) were purchased from Vital River Experimental Animal Services (Beijing, China). All animals were maintained in the animal house controlled at a constant temperature of 23 ± 2°C and a relative humidity of 60–65% with a 12 h light-dark cycle. Water and standard laboratory food were freely available to the animals. All experiments were performed in compliance with the guideline for the care and use of laboratory animals approved by the Animal Ethics Committee of Shanghai University of Tradition Chinese Medicine on October 22, 2014 with the approval number SZY 2014031.

### The Antihypertensive Effect of PASE in SHR After Single Administration

After 1 week of acclimation, 18 SHR animals were randomly divided into 3 groups: “SHR” as the negative control group, “SHR-FOS” as the positive control group, and “SHR-PASE” as the experimental treatment group (*n* = 6/group). Rats in SHR-FOS and SHR-PASE were subjected to single oral administration of 2.5 mg/kg of fosinopril and 400 mg/kg of PASE, respectively. The SHR group was administrated with corresponding volume of distilled water. The systolic, diastolic, mean blood pressure, and heart rate at 0, 2, 4, 6, 8, 12, and 24 h post-dose were measured by the tail-cuff method with non-invasive blood pressure measurement system from Shanghai Alcott Biotech Co., Ltd. (Shanghai, China). Each rat was placed on a 35°C warmer plate and allowed to stabilize for 20 min before measurement. For each time point, at least three values were tested and averaged.

### The Antihypertensive Effect of PASE in SHRs After Continuous Administration

#### Animal Treatment and Antihypertensive Effect Test

Eighteen spontaneously hypertensive rat animals were randomly separated into 3 groups (SHR, SHR-FOS and SHR-PASE, *n* = 6/group) with the daily administration by oral gavage of distilled water, 2.5 mg/kg of fosinopril, and 400 mg/kg of PASE for 12 weeks, respectively. 6 WKY were used as the normal control and treated with distill water. During the administration period, the rats were weighted every 4 days. The systolic, diastolic, mean blood pressures, and heart rates were measured 2 h after administration for every 3 weeks using non-invasive blood pressure measurement system.

#### Biochemical Parameters

At the end of the 12-week treatment, rats were transferred into individual metabolism cages for 24 h to collect urine. Urine samples were centrifuged at 3000 rpm for 15 min to measure the concentrations of urinary total protein. All rats were then anesthetized with intraperitoneal injection of urethane and sacrificed immediately. Blood samples were taken from the abdominal aorta and centrifuged for 10 min at 3000 rpm at 4°C. The supernatant was collected to determine the biochemical parameters reflecting kidney function, electrolyte, and blood pressure condition. Serum urea nitrogen, serum creatinine, and urine total protein were detected using a Hitachi 7080 automatic biochemical analyzer (Hitachi High-Technologies Co., Ltd., Shanghai, China). Serum sodium, potassium, and chloride concentrations were measured using DSI-930B electrolytic analyzer (Shanghai Xunda Medical Instrument Co., Ltd., Shanghai, China). Serum concentrations of angiotensin I (Ang I), angiotensin II (Ang II), and aldosterone (ALD) were determined according to the manufacturer’s instruction of the commercial ELISA Kit obtained from Nanjing Jiancheng Institute of Biotechnology (Nanjing, China).

#### Target Organ Indices

The target organs of each rat, including the heart and 7 cm-long thoracic aorta were removed from connective tissues after sacrificing the animals, then washed by physiological saline and weighed. Left ventricle was divided from the heart through the septum, and the weight and thickness of left ventricle were measured. The organ index was defined as the ratio of the weight (mg) or thickness (μm) of the organ tissue to the body weight (g) of each animal.

#### Histological Examination

The thoracic aortal and left ventricular samples from WKY, SHR, and SHR-PASE groups were fixed in 10 % formalin and then paraffin-embedded for H&E or Masson staining. The sections were examined under light microscope at ×200 magnification using the Olympus image analysis software system (Olympus America, Center Valley, PA, United States). The percentage of collagen in the aortal space, myocardial interstitial space, or around the small artery of left ventricle was calculated using Image-pro Plus 6.0 software (Media Cybernetics, Inc., Rockville, MD, United States).

### ACE Inhibitory Activity Test *in vitro*

The *in vitro* ACE inhibitory activities of PASE (200 μg/mL) and main compounds (2 mM) were performed according to the reported method (Li et al., 2015) using Waters Acquity UPLC system (Waters Corp., Milford, MA, United States) coupled with Micromass Quattro Premier XE tandem quadruple mass spectrometer (Waters Corp., Manchester, United Kingdom) by measuring the content changes of the substrate (hippuryl-histidyl-leucine, HHL) and the product (hippuric acid, HA). The percentage of ACE inhibition was calculated as the following equation: ACE inhibition (%) = $\frac{\text{C}0-\text{C}}{\text{C}0}$ × 100% (C0, HA concentrations before incubation; C, HA concentrations after incubation). Captopril (100 nM) was used as a positive control in the ACEI screening.

### Statistical Analysis

Statistical analysis was performed using SPSS 16.0 statistical software and all data were presented as means ± standard error ($\bar{x}$ ± SE). Body weights, blood pressures, and heart rates were performed by two-way repeated-measures ANOVA with between- (different groups) and within- (different time points) subject factors, and data in each time point were compared by one-way multivariate ANOVA (Bonferroni *post hoc* test). Other data were evaluated by one-way ANOVA with Bonferroni *post hoc* test. Means labeled without a common lowercase letter were considered significantly different at *p* < 0.05.

## Results

### Chemical Constituents in the *Plantago asiatica* L. Seeds Extract

The chemical composition of PASE was determined by ultra-performance liquid chromatography-mass spectrometry (UPLC-MS) in positive ionization scan mode and negative ionization scan mode (Supplementary Figure S1). The peaks of nine major compounds, including phenylethanoid glycosides (isoacteoside, plantamajoside, and acteoside), flavonoids (kaempferol, luteolin, eriodictyol, and isorhamnetin), iridoids (geniposidic acid), and alkaloids (plantagoaminic acid A), were identified by comparing their UPLC retention time and MS spectra to standard compounds.

### Effect of PASE on Body Weight

Spontaneously hypertensive rat had a lower body weight than WKY as previously reported (Kokubo et al., 2005). During 12-week administration, the body weights of the rats in each group increased regularly as shown in Supplementary Figure S2. At the end of the treatment, the body weights of SHR animals showed no significant differences among SHR, SHR-FOS, and SHR-PASE groups.

### Effect of PASE on Blood Pressure and Heart Rate

**FIGURE 1:**
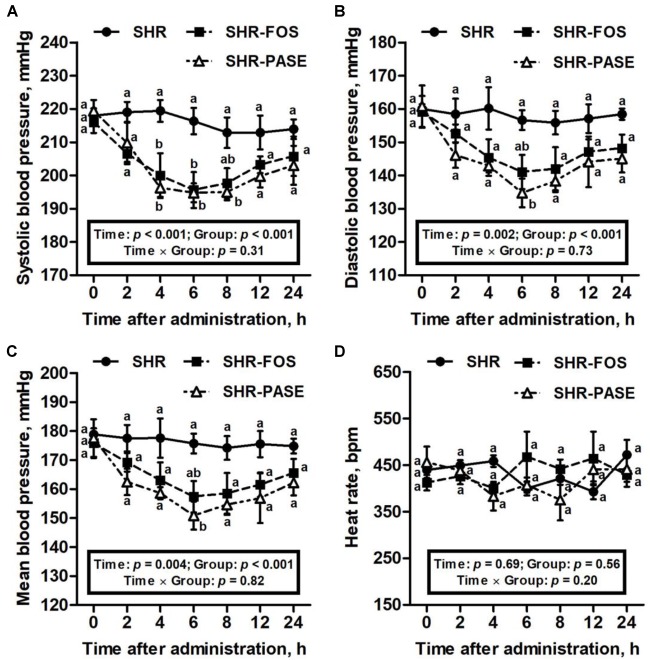
Effect of PASE on blood pressure and heart rate after single administration. Systolic **(A)**, diastolic **(B)**, mean blood pressure **(C)**, and heart rat **(D)** changes of SHR after being singly administrated with PASE. Data are presented by means ± SE; *n* = 6 per group. Blood pressures and heart rates were analyzed by two-way ANOVA with between (groups) and within (hours) subject factors, and data on each time point were by one-way MANOVA with Bonferroni *post hoc* test. Labeled means without a common letter differ indicated a significant difference, *p* < 0.05 (a > b) between groups.

After single administration with 400 mg/kg of PASE, the systolic blood pressure values in SHR were significantly decreased at 4 h and continued to decrease within 8 h as shown in Figure 1. The lowest level of systolic blood pressure in SHR after single treatment of PASE reached to 195 ± 2.84 mmHg occurred at 6 h, which showed a 9.96% (*p* < 0.01) decrease compared with the value without treatment. The diastolic and mean blood pressure results of rats had the similar trend as the systolic blood pressure. After single administration, PASE notably decreased the diastolic and mean blood pressures to 86.0% (*p* < 0.01), and 85.9% (*p* < 0.01) of pressure in SHR group at 6 h, with the values of 135 ± 4.31 mmHg and 151 ± 4.86 mmHg, respectively. However, single administration of PASE did not affect heart rate in SHR animals.

**FIGURE 2:**
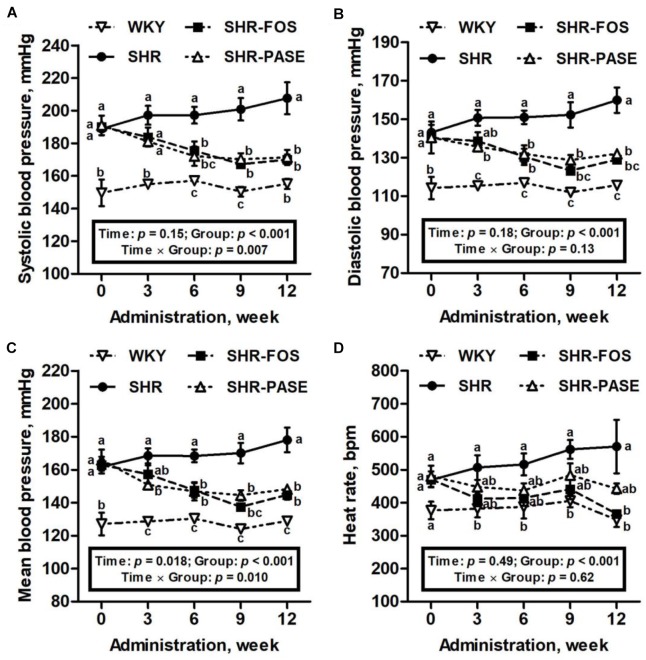
Effect of PASE on blood pressure and heart rate after continuous administration. Systolic **(A)**, diastolic **(B)**, mean blood pressure **(C)**, and heart rate **(D)** changes of SHR after 12-week administration of PASE. Data are presented by means ± SE; *n* = 6 per group. Blood pressures and heart rates were analyzed by two-way ANOVA with between (groups) and within (weeks) subject factors, and data on each time point were by one-way MANOVA with Bonferroni *post hoc* test. Labeled means without a common letter indicated a significant difference at *p* < 0.05 (a > b > c) between groups.

As illustrated in Figure 2, hypertensive animals showed a progressive increase in blood pressure values during the 12-week experimental period from 189 ± 2.35 mmHg to 208 ± 9.79 mmHg. Daily oral administration of PASE induced a remarkable antihypertensive effect on SHR animals. Starting from 6 weeks after treatment, the systolic blood pressures of SHR-PASE group were decreased to 87.2% (6 weeks, *p* < 0.01), 84.8% (9 weeks, *p* < 0.001) and 82.5% (12 weeks, *p* < 0.001) of pressures in SHR group, respectively. Meanwhile, PASE treatment significantly reduced the diastolic blood pressures to 90.1% (3 weeks, *p* < 0.05), 87.4% (6 weeks, *p* < 0.01), 84.6% (9 weeks, *p* < 0.001), and 82.6% (12 weeks, *p* < 0.001) of SHR group, and the mean blood pressures to 89.6% (3 weeks, *p* < 0.05), 87.2% (6 weeks, *p* < 0.01), 84.9% (9 weeks, *p* < 0.001) and 83.2% (12 weeks, *p* < 0.001) of SHR group, respectively. However, no significant difference in blood pressure results were observed between the SHR-PASE and SHR-FOS group. Increasing in heart rates of SHR group were observed during the experimental period. In contrast, PASE lowered the heart rate values by 22.4% compared with SHR group at the end of the treatment, although such reduction in heart rate was not significant (*p* = 0.199).

### Effect of PASE on Target Organ Hypertrophy and Fibrosis

**FIGURE 3:**
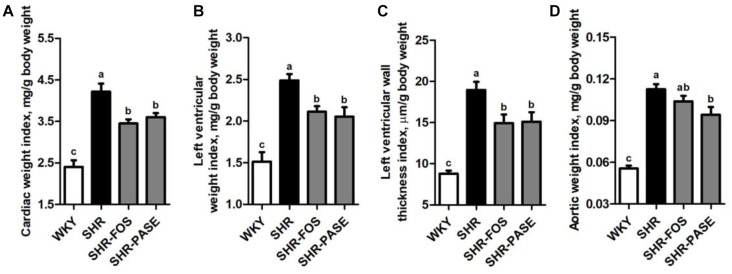
Effect of PASE on target organ indices. Cardiac weight index **(A)**, left ventricular weight index **(B)**, left ventricular wall thickness index **(C)**, and aortic weight index **(D)** of SHR after PASE treatment for 12 weeks. Data are presented by means ± SE; *n* = 6 per group. Statistical analyses were performed by one-way ANOVA with Bonferroni *post hoc* test. Labeled means without a common letter indicated a significant difference at *p* < 0.05 (a > b > c) between groups.

In order to investigate the therapeutic effects of long-term PASE administration on the hypertrophy of target organs, the cardiac weight index, left ventricular weight index, left ventricular wall thickness index, and aortic weight index were measured. Hypertension is known to induce remarkable hypertrophy of cardiac and arterial tissues in SHR animals. After 12-week treatment of PASE, SHR animals in SHR-PASE group displayed obvious improvements of the target organ hypertrophy, which decreased to 85.4% (*p* < 0.05), 82.6% (*p* < 0.01), 79.7% (*p* < 0.05), and 83.7% (*p* < 0.05) as compared to SHR group in cardiac weight index, left ventricular weight index, left ventricular wall thickness index, and aortic weight index, respectively (Figure 3).

**FIGURE 4:**
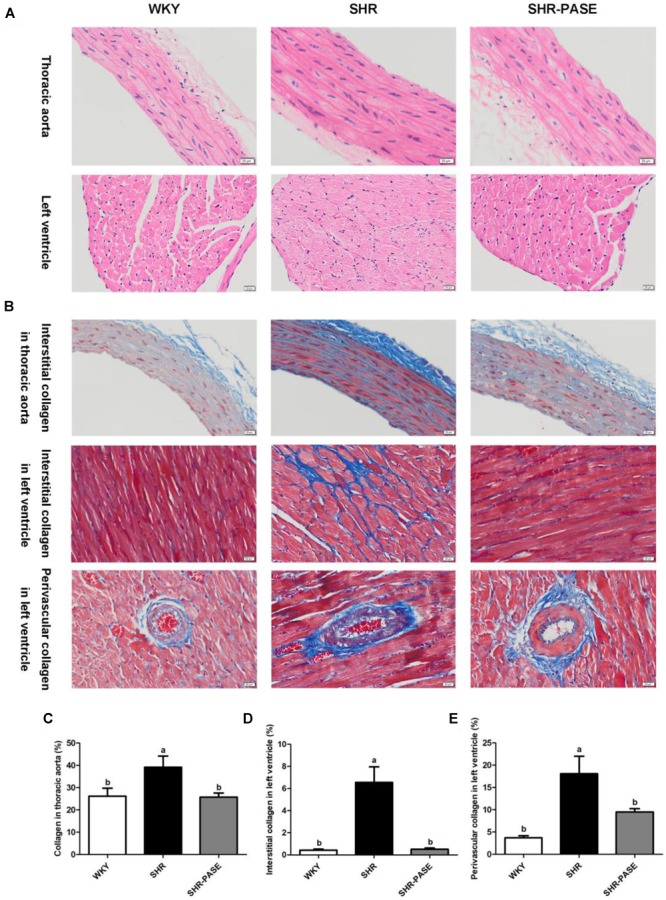
Effect of PASE on target organ hypertrophy and fibrosis. Histology of thoracic aortal and left ventricular tissues from SHR after 12-week administration of PASE. H&E staining **(A)** were used to investigate the morphological changes and Masson staining **(B)** to examine fibrosis levels in the target organs (original magnification × 200). Contents of collagen in thoracic aorta **(C)**, myocardial interstitial **(D)**, and perivascular spaces **(E)** of left ventricle were also measured. Data are presented by means ± SE; *n* = 6 per group. Statistical analyses were performed by one-way ANOVA with Bonferroni *post hoc* test. Labeled means without a common letter indicated a significant difference at *p* < 0.05 (a > b) between groups.

Meanwhile, the histological changes of heart and aorta tissues were determined among WKY, SHR, and SHR-PASE groups. Representative H&E staining images of each group were shown in Figure 4A. The left ventricular and thoracic aortic tissues showed that degeneration and hypertrophy occurred in myocardia and vascular smooth muscle cells of SHR group compared to WKY group. After treatment with PASE, alleviated degeneration and hypertrophy in target tissues were observed in SHR animals. The collagen contents in left ventricular and thoracic aortic tissues were measured by Masson staining method to investigate the fibrosis degrees of the target organs. Compared with WKY group, SHR group showed significant more collagen accumulation in target tissues (Figure 4B), while PASE treatment reduced the collagen contents in thoracic aorta (Figure 4C), myocardial interstitial (Figure 4D), and perivascular spaces of left ventricle (Figure 4E) to 65.8% (*p* < 0.05), 7.67% (*p* < 0.001), and 52.7% (*p* < 0.05) of SHR group, respectively.

### Effect of PASE on Biochemical Parameters of Kidney Function and Electrolyte

**FIGURE 5:**
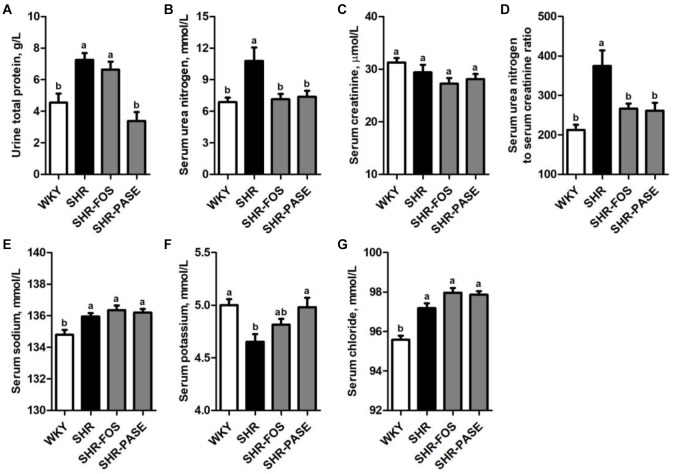
Effect of PASE on biochemical parameters of kidney function and electrolyte. Total protein in urine **(A)**; urea nitrogen **(B)**; creatinine **(C)** in serum; the ratio of serum urea nitrogen and creatinine **(D)**; serum sodium **(E)**; potassium **(F)**; and chloride **(G)** concentrations in SHR after PASE treatment for 12 weeks. Data are presented as means ± SE; *n* = 6 per group. Statistical analyses were performed by one-way ANOVA with Bonferroni *post hoc* test. Labeled means without a common letter indicated a significant difference at *p* < 0.05 (a > b) between groups.

Urine total protein, serum urea nitrogen and serum creatinine were measured to investigate the effects of PASE on kidney function. As shown in Figure 5, the levels of urine total protein and serum urea nitrogen in SHR group were remarkably increased by 59.7% (*P* < 0.01) and 57.0% (*P* < 0.01), compared to WKY group, and these elevated biomarker levels were depressed by 53.3% (*P* < 0.001) and 31.6% (*P* < 0.05) in SHR-PASE group when animals were orally administrated with PASE. Serum creatinine did not show obvious changes before and after PASE treatment. The ratio of serum urea nitrogen to serum creatinine showed significantly increase by 76.1% (*P* < 0.001) in SHR group as compared to WKY group and were reduced by 30.3% (*P* < 0.01) in SHR-PASE group as compared to SHR group.

Serum electrolyte levels, including sodium, potassium and chloride, were also determined. The serum potassium was remarkably raised to 4.98 mmol/L after treatment with PASE, which was 4.65 mmol/L in the model rats. The serum sodium and chloride were not significantly affected by PASE (Figure 5).

### Effect of PASE on Renin-Angiotensin-Aldosterone System

**FIGURE 6:**
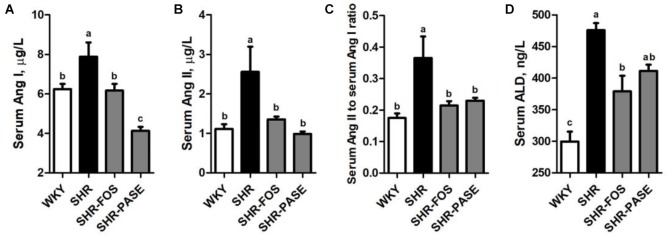
Effect of PASE on renin-angiotensin-aldosterone system. Serum Ang I **(A)** and Ang II **(B)**; the ratio of serum Ang II and Ang I **(C)**; and serum ALD **(D)** concentrations in SHR after PASE treatment for 12 weeks. Data are presented as means ± SE; *n* = 6 per group. Statistical analyses were performed by one-way ANOVA with Bonferroni *post hoc* test. Labeled means without a common letter indicated a significant difference at *p* < 0.05 (a > b > c) between groups.

The concentrations of Ang I, Ang II, and ALD have been used as important biological parameters to evaluate the function of RAAS which is closely related to hypertension. As shown in Figure 6, the serum Ang I and Ang II in SHR group showed remarkably increase by 26.4% (*p* < 0.05) and 130.6% (*p* < 0.01) compared to WKY group, respectively, while SHR-PASE group produced significant lower levels in these two parameters. The concentration ratio of serum Ang II to Ang I was also measured to partly present the activity of angiotensin-converting enzyme (ACE), which showed significant decrease in SHR-PASE animals (*p* < 0.05). The serum ALD value were tested and showed a decrease after PASE treatment (*p* = 0.265).

### *In vitro* ACE Inhibition Test of PASE and Active Compounds Screening

**FIGURE 7:**
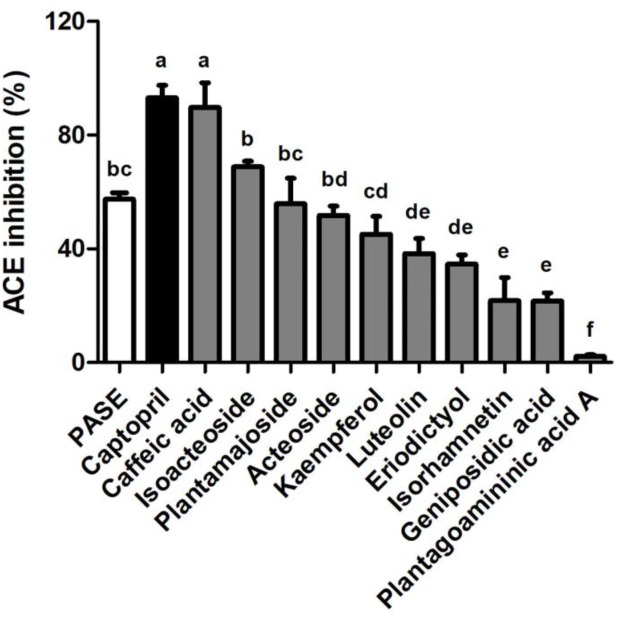
*In vitro* ACE inhibition test of PASE and active compounds screening. Inhibitory activities on ACE of PASE and major components (*n* = 3). Statistical analyses were performed by one-way ANOVA with Bonferroni *post hoc* test. Labeled means without a common letter indicated a significant difference at *p* < 0.05 (in order of a to f) between groups.

We used a validated incubation-UPLC-MS/MS approach (Li et al., 2015) to confirm the ACE inhibition potency of PASE by quantifying the production of HA from HHL. Captopril (100 nM) was used as a positive control in the ACEI screening. At 200 μg/mL, PASE showed potency of ACE inhibitory greater than 50%. 9 major compounds in PASE were identified to belong to phenylethanoid glycosides (isoacteoside, plantamajoside, and acteoside), flavonoids (kaempferol, luteolin, eriodictyol, and isorhamnetin), iridoids (geniposidic acid), alkaloids (plantagoaminic acid A) and they were further screened for ACE inhibition, As shown in Figure 7, these compounds exhibited a range of inhibition on ACE from 2.19 to 68.82% at the same concentration (2 mM) in the following order: caffeic acid, isoacteoside, plantamajoside, acteoside, kaempferol, luteolin, eriodictyol, isorhamnetin, geniposidic acid, and plantagoaminic acid A. Caffeic acid, as one of the major metabolites of isoacteoside, plantamajoside and acteoside, had the strongest inhibitory activity on ACE among all compounds.

## Discussion

This work was focused on investigating the therapeutic property of the extract of *Plantago astica* L., a folk medicine widely used in Asian countries against hypertension. SHR is used in this work, which is considered as a common animal model for the study of essential hypertension in human (Pinto et al., 1998). Firstly, PASE showed significant antihypertensive effects after single and long-term intragastric administration in SHR. Secondly, PASE treatment effectively ameliorated cardiac hypertrophy and vascular remodeling, and exerted protective effect on kidney. Finally, the inhibitory activity on ACE *in vitro* with the major active constituents of phenylethanoid glycosides were probably involved in the antihypertensive effects of PASE.

Previous studies found that *P. asiatica* L. seeds and its compound prescriptions were applied to treat hypertension clinically due to its diuresis (Doan et al., 1992; Chou et al., 2018). The diet supplemented with *Plantago ovata* husks prevented the development of hypertension in Obese Zucker Rats (Galisteo et al., 2005). In the present study, the blood pressure values, including systolic, diastolic and mean blood pressures were remarkably lowered at 4 h after acute administration of PASE and lasted 4 more hours Furthermore, the blood pressure values were decreased after 3 weeks of continuous administration and significant differences between the PASE treated group and the water treated group were observed since 6 weeks of treatment, and such differences were growing greater to the end of the experiment. However, PASE treatment had no significant effect on heat rate in acute and choric treatment or caused any reduction on body weight in choric treatment experiment, suggesting the adequate safety for PASE treatment.

The primary pathological changes of hypertension were left ventricular hypertrophy (LVH) (Devereux et al., 1987), which was characterized by an increase in myocyte size and fibrosis, and vascular remodeling (Heagerty et al., 1993). Studies suggested cardiac hypertrophy was viewed as the main target for the treatment of hypertension (Zanchetti, 2010). The compensatory thickening of the left ventricular wall was existed to normalize wall stress from hypertension in SHR (Mitchell et al., 1997). Meanwhile, studies demonstrated that the vessels in SHR were remodeled by the decreased lumen and increased media (Heagerty et al., 1993; Intengan and Schiffrin, 2001). In the present study, we confirmed LVH and an inward remodeling in thoracic aorta of SHR compared with WKY. PASE treatment not only reduced target organ indexes but also alleviated collagen contents in left ventricular and thoracic aortic tissues.

Numerous studies indicated that chronic high blood pressure caused renal disease, evidenced by tubular atrophy, interstitial fibrosis, and glomerular alterations (Goldsmith and Hamilton, 1998; Tomiyama et al., 2014). Urea protein was regarded as a major risk factor of renal disease progression and a crucial pathogenic role in renal dysfunction (Cravedi and Remuzzi, 2013). Blood urea nitrogen and serum creatinine were unable to indicate structural renal disease alone, while the ratio of blood urea nitrogen to serum creatinine could be as an evaluation index for the confirmation of renal functional and structural integrity (Baum et al., 1975). In our study, PASE was found to notably reduce the high levels of urine total protein, serum urea nitrogen and serum urea nitrogen to serum creatinine ratio in SHR. It is well recognized that water and electrolyte homeostasis, namely the transport and excretion of sodium, potassium and chloride, were closely related to renal function (Wirz and Geigy, 1961; Gavras et al., 1976). Previous researches indicated that potassium supplementation effectively ameliorated high blood pressure and renal injury in SHR (Galdino et al., 2010; Jodas et al., 2014). The results in our present study demonstrated long-term intake of PASE increased the serum levels of K+. In a nutshell, our findings manifested PASE had the protective effect on renal function against hypertension.

The RAAS plays an important role in regulating the homeostasis of body fluids, electrolytes and blood pressure (Gavras et al., 1976). Renin, a proteinase, was released from the kidney, which activated Angiotensinogen transformation into inactive Angiotensin I (Ang I) in blood circulation. Further, Ang I was degraded to Angiotensin II (Ang II) by ACE (Erdös, 1976). In the RAAS, Ang II was one of the most powerful vasoconstrictive compounds. It binds to Angiotensin II receptor type 1 (AT1) to shrink capillary, regulate aldosterone (ALD) secretion and remodel myocardium and vessel, which lead to high blood pressure and cardiac dysfunction. In that case, abnormal activation of RAAS will cause hypertension and related cardiovascular disorders. In our study, the down-regulated Ang I, Ang II, Ang II to Ang I ratio, and ALD in PASE treated groups suggested that the antihypertensive effect of PASE could be achieved by regulating the RAAS.

In the RAAS, ACE is a key protease to produce Ang II. Hence, inhibition of ACE is considered a major therapeutic approach in the treatment of hypertension and cardiovascular disease. In recent years, many plant components, Ocimum gratissimum extract (Shaw et al., 2017), mango leaf Extract (Ronchi et al., 2015), Carica papaya extract (Brasil et al., 2014), and mulberry leaf aqueous extract (Yang et al., 2012), had shown anti-hypertension effects through an inhibition of ACE activity. According to our previous research on ACE inhibition of phenylethanoid glycosides from *P. asiatica* L., the IC50 values ranged from 0.53 to 15.035 mM (Li et al., 2015). In this study, inhibition of ACE by various active components of PASE were tested *in vitro* at 2 mM. Consistent with other published reports (Geng et al., 2010), we found PASE and its major constituents of 8 was concurrently aligned with the reduction of serum Ang II, Ang II to Ang I ratio, and ALD in PASE treated SHR. However, there are many kinds of compounds in TCM extracts, and the absorption or distribution is greatly complicated *in vivo*. The blood concentration of active compounds, the interaction of compounds and the activity of metabolites after oral administration of PASE *in vivo* needs to be investigated in the future, which will provide the basis for the experiment *in vitro.*

In conclusion, we have demonstrated that PASE has the long-acting anti-hypertension effect and ACE inhibition may be the mechanism of PASE antihypertensive activity. Phenylethanoid glycosides including isoacteoside, plantamajoside and acteoside, might be considered as major active compounds of PASE for the antihypertensive effect in SHRs. Meanwhile, PASE also protects organ injury including LVH, vascular remodeling and renal dysfunction. Our results suggest that PASE may be a potential therapy for blood pressure controlling and cardiovascular disease prevention.

## Author Contributions

MQ, Q-MY, Z-TW, and LY developed the conception and design of the study. R-CT, MQ, Q-MY, P-FL, D-DW, and J-PL performed the experiments and analyzed the data. Z-TW and LY revised the manuscript. All authors contributed to the final manuscript and approved it for publication.

## Conflict of Interest Statement

The authors declare that the research was conducted in the absence of any commercial or financial relationships that could be construed as a potential conflict of interest.
