# Supplementary material for: Extract of Plantago asiatica L. Seeds Ameliorates Hypertension in Spontaneously Hypertensive Rats by Inhibition of Angiotensin Converting Enzyme
Source: Front Pharmacol. 2019 Apr 30;10:403. doi: 10.3389/fphar.2019.00403 (PMC6502967; doi:10.3389/fphar.2019.00403)
Supplement: Supplementary file 1 [file Data_Sheet_1.PDF]

Supplemental Text and Figures

Extract of Plantago asiatica L. seeds ameliorates hypertension in spontaneously hypertensive rats via inhibition of angiotensin converting enzyme

Ren-Chao Tong[1†], Meng Qi[1†], Qi-Ming Yang[1], Peng-Fei Li[1], Dan-Dan Wang[1], Ji-Ping Lan[1, 2], Zheng-Tao Wang[1]*, Li Yang[1]*

[1]The Ministry of Education (MOE) Key Laboratory for Standardization of Chinese Medicines and the State Administration of Traditional Chinese Medicine (SATCM) Key Laboratory for New Resources and Quality Evaluation of Chinese Medicines, Institute of Chinese Materia Medica, Shanghai University of Traditional Chinese Medicine, Shanghai 201203, China
[2]Institute of Interdisciplinary Integrative Medicine Research, Shanghai University of Traditional Chinese Medicine, Shanghai 201203, China

[*] Correspondence:
ztwang@shutcm.edu.cn (Z.W.); yangli7951@hotmail.com (L.Y.);

[†] The authors contributed equally to this work.

Running title: Antihypertensive effect of Plantago asiatica L. seeds extract

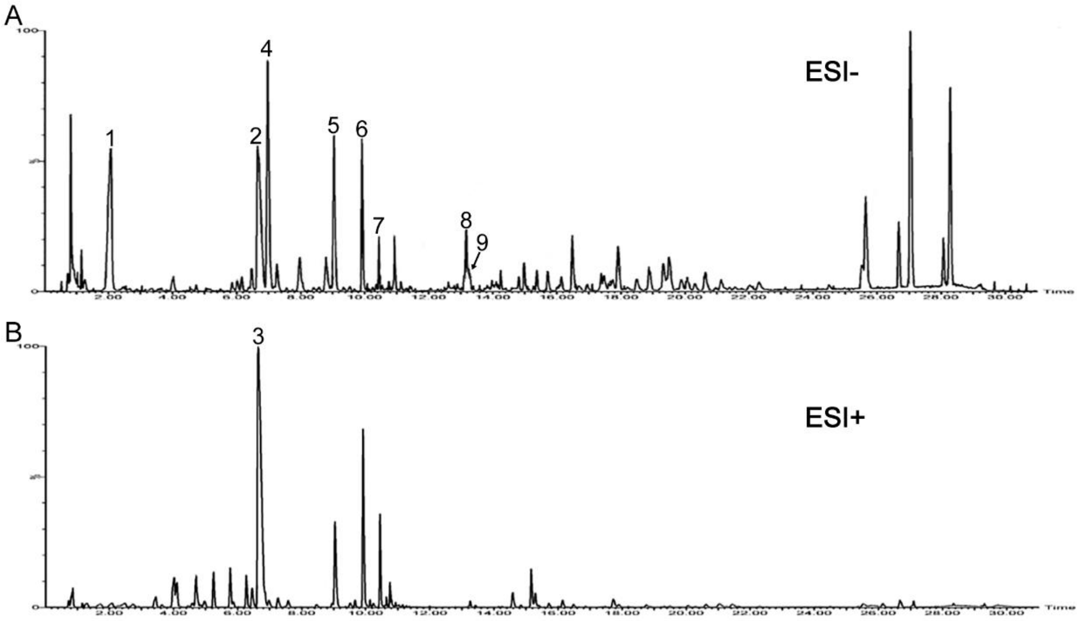

Figure S1. Base peak ion chromatograms of PASE obtained by UPLC–ESI-QTOF-MS. (A) Chromatogram in negative ionization mode; (B) chromatogram in positive ionization mode. 1. Geniposidic acid; 2. Plantamajoside; 3. Plantagoamininic acid A; 4. Acteoside; 5. Isoacteoside; 6. Eriodictyol; 7. Luteolin; 8. Kaempferol; 9. Isorhamnetin. These compounds are confirmed by standards.

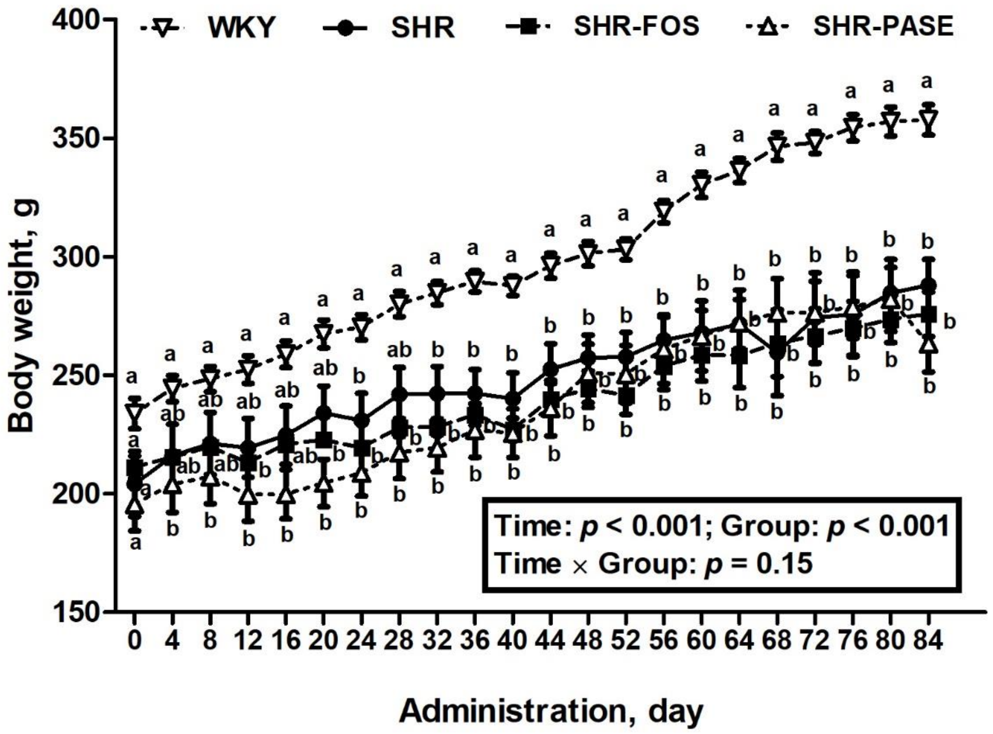

Figure S2. Effect of PASE on body weight.
Body weight changes of rats during 12-week treatment of PASE. Data are presented by means ± SE; n = 6 per group. Data are presented by means ± SE; *n* = 6 per group. Body weights were analyzed by two-way ANOVA with between (groups) and within (days) subject factors, and data on each day were by one-way MANOVA with Bonferroni post hoc test. Labeled means without a common letter differ at $p < 0.05$ (a > b > c).
